# Letrozole cotreatment with progestin-primed ovarian stimulation in women with polycystic ovary syndrome undergoing IVF treatment

**DOI:** 10.3389/fphys.2022.965210

**Published:** 2022-08-19

**Authors:** Yali Liu, Jiaying Lin, Li Chen, Xiaoyan Mao, Li Wang, Qiuju Chen, Sha Yu, Yanping Kuang

**Affiliations:** Department of Assisted Reproduction, Shanghai Ninth People’s Hospital Affiliated to Shanghai Jiaotong University School of Medicine, Shanghai, China

**Keywords:** letrozole, progestin-primed ovarian stimulation, polycystic ovarian syndrome, *in vitro* fertilization, frozen embryo transfer

## Abstract

**Background:** Progestin is an alternative to gonadotropin-releasing hormone (GnRH) analogues in the follicular phase to suppress the premature luteinizing hormone (LH) surge in women with polycystic ovary syndrome (PCOS). However, progestin-primed ovarian stimulation (PPOS) is always accompanied by increased pituitary suppression and gonadotropin consumption. Previous studies suggested that letrozole appeared to have the potential to reduce the total gonadotropin dose required for ovarian stimulation. A retrospective cohort study was performed to evaluate the efficacy of PPOS with or without letrozole in infertile women with PCOS.

**Methods:** This retrospective cohort study included 448 women with PCOS who underwent controlled ovarian stimulation (COS) with human menopausal gonadotropin (hMG) and medroxyprogesterone acetate (MPA) (n = 224) or hMG and MPA cotreatment with LE (n = 224) from January 2018 to March 2021 after propensity-score matching. The primary outcome measure was the hMG dose. The secondary outcomes were the durations of ovarian stimulation, the implantation rate, the number of oocytes retrieved and viable embryos, oocyte maturity and fertilization rates, the percentage of women with profound pituitary suppression (luteinizing hormone [LH] <1.0 IU/L on the trigger day).

**Results:** The hMG doses (1949.89 ± 725.03 IU vs 2017.41 ± 653.32 IU**,** *p* > 0.05) and durations of ovarian stimulation (9.03 ± 1.79 days vs 9.21 ± 2.18 days**,** *p* > 0.05) were similar between the two groups. The implantation rate was significantly higher in the study group (MPA + hMG + LE) than in the control group (MPA + hMG) (42.22 vs 34.69%, *p* < 0.05). The numbers of oocytes and embryos retrieved were similar between the two groups. Interestingly, letrozole cotreatment was associated with decreased oocyte maturity and fertilization rates in comparison with standard PPOS protocols even though mature and fertilized oocyte yields were comparable. Compared with those in the control group, the LH values on the trigger day were significantly higher in the study group, together with significantly reduced pituitary suppression.

**Conclusion:** Letrozole combined with PPOS cannot reduce hMG consumption in PCOS patients undergoing IVF treatment and shows no beneficial effect on cycle characteristics of COS. However, letrozole supplementation manifests as a superior implantation rate to that of the standard PPOS protocol in women with PCOS.

## Introduction

Polycystic ovary syndrome (PCOS) is a highly prevalent endocrine and reproductive disorder, and more than 80% of patients with anovulatory infertility suffer from PCOS (Balen et al., 2016). *In vitro* fertilization (IVF) serves as a third-line treatment for infertile PCOS patients who fail to respond to lifestyle modification or ovulation induction (OI) therapies (Thessaloniki, 2008). The conventional protocol for ovarian stimulation with exogenous gonadotropin (Gn) and gonadotropin-releasing hormone (GnRH) analogues can support multifollicular development and inhibit a premature luteinizing hormone (LH) surge. However, attaining consistent pituitary suppression is complex and carries a relatively high risk of ovarian hyperstimulation syndrome (OHSS) due to human chorionic gonadotropin (hCG) injection (Macklon et al., 2006). Compared with normal ovulatory women, patients with PCOS more frequently face problems such as OHSS, poor oocyte quality, fertilization failure and pregnancy complications (Qiao and Feng, 2011; Timmons et al., 2019).

Progestin-primed ovarian stimulation (PPOS), which administers medroxyprogesterone acetate (MPA) and human menopausal gonadotropin (hMG) from the early follicular phase, can effectively prevent the oestradiol (E2)-induced LH surge and thus serves as an alternative to conventional GnRH analogues (Kuang et al., 2015). Progestin can reduce the incidence of moderate or severe OHSS without adversely affecting clinical outcomes in patients with PCOS (Wang et al., 2016). Nevertheless, the use of MPA in the PPOS regimen tends to inhibit the pituitary in a more profound manner and therefore requires higher doses of gonadotropin (Gn) than the use of GnRH analogues in patients with PCOS (Wang et al., 2016). Thus, optimizing the PPOS protocol to make it more economical, efficient, and safer is an avenue we have been trying to explore.

Letrozole (LE) is a nonsteroidal, highly selective oral aromatase inhibitor (AI) that inhibits the synthesis of oestrogen and increases the secretion of endogenous gonadotropin by diminishing negative feedback to stimulate ovulation (Requena et al., 2008). Currently, letrozole is widely used as an adjunct for IVF cycles (Yang et al., 2021). Cotreatment with letrozole during ovarian stimulation for IVF could significantly reduce gonadotropin consumption in normal and poor ovarian responders without decreasing the number of retrieved oocytes, some even accompanied by increased pregnancy outcomes, while few studies have focused on the clinical characteristics of letrozole coadministration in PCOS patients treated for IVF (Bulow et al., 2022; Jiang et al., 2022). The endocrine and ovulation induction characteristics of PCOS patients are different from those of normal and poor ovarian responders. Hence, it remains unclear whether the letrozole combination is beneficial for women with PCOS treated with PPOS. The present retrospective cohort study was designed to investigate the effects of LE combined with the PPOS protocol on the characteristics of the oocyte pick-up cycle and frozen embryo transfer (FET) cycle in PCOS female patients.

## Materials and methods

### Study setting and patients

**FIGURE 1:**
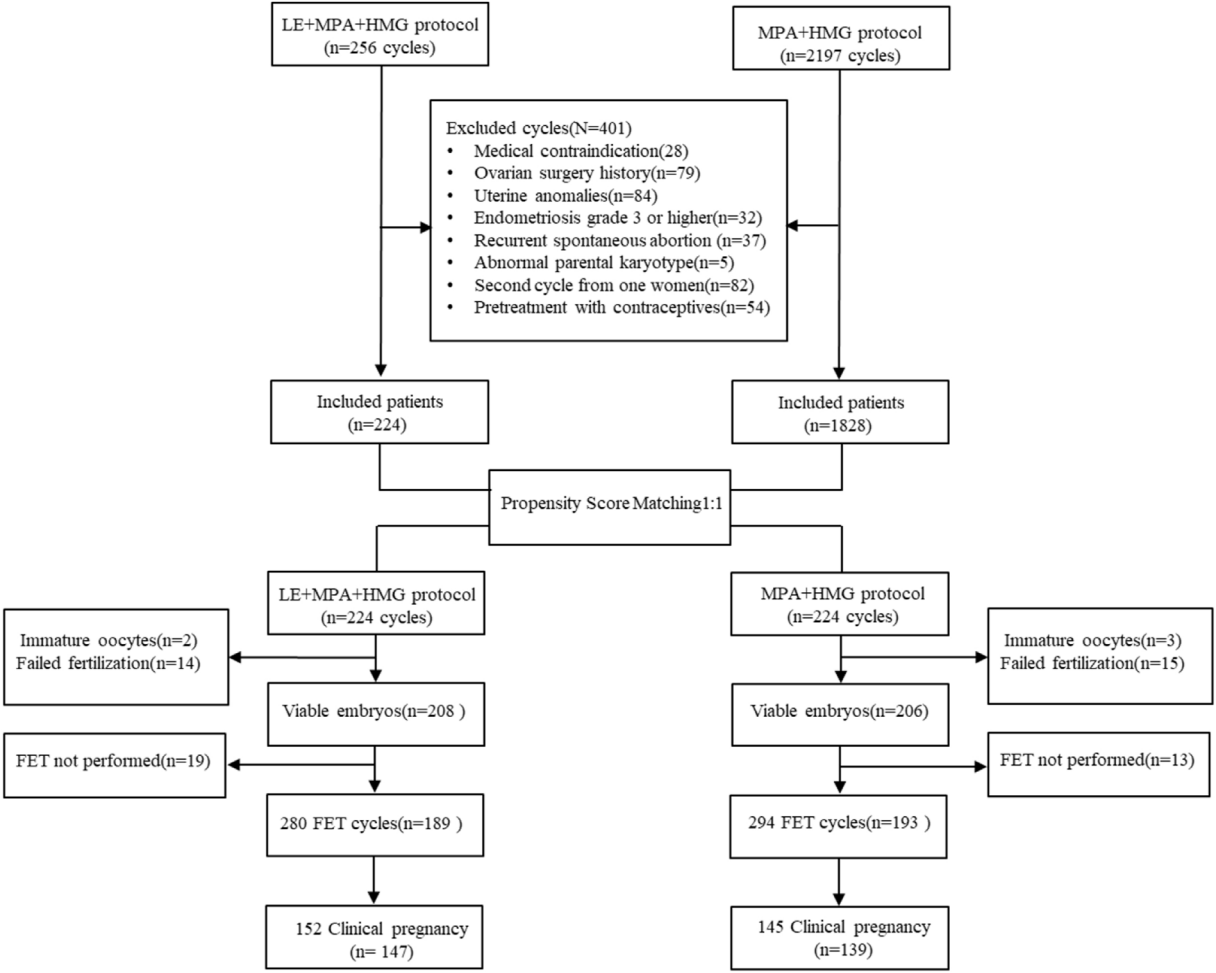
Flow chart of the study. IVF: *In vitro* fertilization; ICSI: Intracytoplastic sperm injection; FET: Frozen embryo transfer; LE: Letrozole; MPA: Medroxyprogesterone acetate; HMG: Meno-trophin for Injection.

Women with PCOS who underwent IVF/ICSI cycles using PPOS (hMG + MPA) or PPOS cotreatment with letrozole (hMG + MPA + LE) from January 2018 to March 2021 were enrolled. This study was approved by the Ethics Committee (Institutional Review Board) of Shanghai Ninth People’s Hospital. Patients met the following criteria: 1. Between 20 and 40 years of age; 2. Basal follicle-stimulating hormone (FSH) level <10 mIU/ml; and 3. No more than one previous nonviable embryo cycle. The diagnosis of PCOS was made according to the 2003 Rotterdam consensus (Rotterdam, 2004) and the recent guidelines for PCOS 2018 (Teede et al., 2018), whereby at least two out of three of the following criteria were met: 1) oligo- and/or anovulation; 2) biochemical and/or clinical evidence of hyperandrogenism; or 3) polycystic ovarian morphology on ultrasound. Where both oligo- or anovulation and hyperandrogenism were present, ultrasound was not necessary for diagnosis. Once diagnosed, its assessment and management covered reproductive, metabolic, and psychological features. The following diseases were excluded: hyperandrogenism and ovulatory dysfunction of other aetiologies, including congenital adrenal hyperplasia, androgen-secreting tumours, hyperprolactinemia and thyroid disease. Women who were on medications for conditions such as diabetes and hypertension were also excluded. A flowchart of the study is shown in Figure 1.

### Controlled ovarian stimulation

From menstrual cycle day 3 (MC3) to the trigger day, patients were administered 150–225 IU/d hMG (Anhui Fengyuan Pharmaceutical Co., China) via intramuscular injection and 4 mg/d MPA (Shanghai Xinyi Pharmaceutical Co., China) via oral administration. Letrozole (Jiangsu Hengrui Medicine Co., China) 2.5 mg/day was given from cycle day 3 onwards for only 5 days in the study group. The starting dose of hMG was 150 IU/day for patients with a high antral follicle count (>20) or slightly elevated basal FSH (7–10 mIU/ml), and a daily dose of 225 IU hMG was given to the other patients. For both groups, the hMG doses were adjusted depending on the number and size of the developing follicles on ultrasound as well as serum concentrations of sex hormones, including FSH, LH, oestradiol (E2) and progesterone (P4), administered beginning on MC8 and every 2–4 days thereafter. The final stage of oocyte maturation was triggered by the combination of 0.1 mg triptorelin (Decapeptyl; Ferring Pharmaceuticals, Germany) and 1000 IU hCG (Lizhu Pharmaceutical Trading Co., China) once the leading follicle exceeded 20 mm or at least three follicles exceeded 18 mm in diameter. Oocyte retrieval was performed 34–36 h after the trigger and guided by transvaginal ultrasound (TVS). All follicles with a diameter over 10 mm were aspirated (Kuang et al., 2015).

After oocyte retrieval, fertilization was carried out *in vitro* by either standard IVF or intracytoplasmic sperm injection (ICSI) according to the semen quality and previous fertilization conditions (Xu et al., 1998). Embryos were examined for the number or regularity of blastomeres and the degree of fragmentation. Top-quality embryos (including grade-1 and grade-2 6-cell embryos and above) were frozen by vitrification within 3 days after oocyte retrieval according to the criteria described by Cummins et al. (Cummins et al., 1986; Kuang et al., 2015). The non-top-quality embryos were placed in extended culture, and blastocysts with good morphology were also frozen on day 5 or 6 (Kuang et al., 2015).

### Hormone measurement

Serum FSH, LH, E2 and P levels were measured on MC3, MC8, MC10-12, the trigger day and the day after trigger. Hormone concentrations were measured by means of chemiluminescence (Abbott Biologicals B.V, the Netherlands). The lower limits of sensitivity were as follows: FSH, 0.06 mIU/ml; LH, 0.09 mIU/ml; E2, 10 pg/ml; and P4 0.1 ng/ml. The upper limit for the E2 measurement was 5,000 pg/ml. If the E2 level on the trigger day or the next day was greater than the upper limit, it was recorded as 5,000 pg/ml without repeating the assay after sample dilution.

### Endometrial preparation and frozen embryo transfer

Endometrial preparation and FET were arranged for the second cycle after oocyte retrieval as previously described (Kuang et al., 2014; Kuang et al., 2015). Mild stimulation with letrozole was first recommended for FET, while hormone replacement therapy (HRT) was conducted for those who failed to become pregnant with mild stimulation cycles or with a history of a thin endometrium (endometrial thickness ≤6 mm). In the mild stimulation cycle, women were administered letrozole 2.5/5 mg for 5 days beginning at MC3. When the dominant follicle was ≥17 mm in diameter with an endometrial lining >8 mm, an E2 level >150 pg/ml and a P4 level <1 ng/ml, a bolus of urinary hCG (5000 IU) was injected for ovulation triggering. Progesterone commenced 2 and 3 days later, followed by day 3 embryo transfer 4 and 5 days later or blastocyst transfer 6 and 7 days later via abdominal ultrasound guidance.

Women who failed pregnant in the first FET cycle routinely underwent hysteroscopy and endometrial curettage, the uterine growth would be removed if they have and send pathology. For patients with intrauterine adhesions, decomposition of adhesions or placement of intrauterine contraceptive ring (IUD) were given according to the severity. If patients have had hysteroscopy within 6 months, the hysteroscopic procedure would not be repeated.

### Outcome measures

The primary outcome measure of this study was the hMG dose. The secondary measures included the durations of ovarian stimulation, the implantation rate in FET cycles, the number of oocytes retrieved, the number of viable embryos, profound LH suppression (a serum LH level less than 1 mIU/ml on the trigger day during ovarian stimulation (Liu et al., 2018). The oocyte retrieval rate was calculated as the total number of oocytes retrieved divided by the total number of follicles punctured; the mature oocyte rate was calculated as the total number of mature oocytes divided by the total number of oocytes retrieved; the fertilization rate was calculated as the total number of fertilized oocytes divided by the total number of mature oocytes; and the cleavage rate was calculated as the total number of cleaved embryos divided by the total number of fertilized oocytes. The clinical pregnancy rate, miscarriage rate, and ectopic pregnancy rate was recorded. The viable embryo rate per oocyte was defined as the number of viable embryos divided by the number of oocytes retrieved. The cycle cancellation rate was estimated based on the number of patients who had no viable embryos after complete oocyte retrieval. The implantation rate was calculated according to the number of gestational sacs divided by the number of embryos transferred. Clinical pregnancy was defined as the presence of at least one gestational sac with or without foetal heart activity on ultrasound examination at 4 weeks after FET, and the clinical pregnancy rate was the number of clinical pregnancies divided by the number of FET cycles. The miscarriage rate was the proportion of pregnancies that ended in spontaneous or therapeutic abortion.

### Statistical analysis

A propensity score matching (PSM) model was established to balance differences in baseline characteristics between the two groups. To estimate the propensity score, we selected 10 covariates, namely, age; antral follicle count (AFC); basal levels of FSH, LH, E2 and P4; type of infertility (primary or secondary); infertility duration; previous IVF attempts (0, 1–2 or ≥3); and body mass index (BMI). Patients using MPA + hMG + LE were matched with the MPA + hMG group using the nearest-neighbour random matching algorithm at a ratio of 1:1. PSM was conducted using the R statistical programming language (version 4.0.3; R Foundation for Statistical Computing, Vienna, Austria).

The data were evaluated by Student’s *t* test for continuous variables with a normal distribution and the Mann–Whitney *U* test for continuous variables with a nonnormal distribution. These data are presented as the mean (standard deviation, SD). Pearson’s chi-square test or Fisher’s exact test was used for categorical variables (presented as %), as appropriate. Data analysis was performed with the Statistical Package for the Social Sciences (version 24, SPSS Inc.). Two-sided *p* < 0.05 was considered statistically significant.

## Results

### Patient characteristics

Figure 1 shows a profile summary of the study. Briefly, a total of 2453 PCOS women who were candidates for assisted reproductive technology treatment from January 2018 to March 2021 were enrolled in this study, and 401 cycles were excluded as described in the Materials and Methods section. Of the remaining 2052 patients, 224 patients undergoing the MPA + hMG + LE protocol were matched with 224 patients who underwent the MPA + hMG protocol. All these patients completed oocyte retrieval cycles and succeeded in producing oocytes (range, 1–62), while 34 patients had no viable embryos. Among the remaining patients, 371 women completed FET cycles. No significant between-group differences were found in the postmatching analysis with regard to any baseline characteristics (all *p* > 0.05) (Table 1).

**TABLE 1:** Baseline characteristics of women undergoing IVF/ICSI.

Characteristic	Study group (hMG + MPA + LE; n = 224)	Control group (hMG + MPA; n = 224)	*p* Value
**Age (y), mean ± SD**	31.79 ± 3.59	31.79 ± 3.51	0.99
**BMI (kg/m2), mean ± SD**	24.15 ± 4.39	24.34 ± 4.02	0.64
**Duration of infertility (y), mean ± SD**	3.73 ± 2.46	3.42 ± 1.73	0.14
**Primary infertility, n (%)**	66.96 (150/224)	68.31 (153/224)	0.737
**Indication, n (%)**			0.916
**Tubal factor**	135	132	
**Male factor**	45	49	
**Unknown factor**	33	30	
**Combination of factors**	11	13	
**Previous IVF failure, n (%)**			0.343
**0**	195	194	
**1–2**	16	22	
**> 3**	13	8	
**MC3 hormone levels, mean ± SD**			
**FSH (IU/L)**	5.17 ± 1.22	5.17 ± 1.07	0.96
**LH (IU/L)**	5.07 ± 3.29	5.04 ± 3.24	0.91
**E**_**2**_ **(pg/ml)**	34.7 ± 11.54	33.72 ± 11.81	0.38
**P (ng/ml)**	0.25 ± 0.13	0.25 ± 0.11	0.91
**AFC**	20.52 ± 6.05	21.3 ± 7.03	0.21

Data are presented as mean ± standard deviation or number (percentage). BMI, body mass index; MC3, day 3 of the menstrual cycle; FSH, follicle stimulating hormone; LH, luteinizing hormone; E2, estradiol; P, Progester-one; AFC, antral follicle count.

### Ovarian stimulation, follicle development, and oocyte performance

The MPA + hMG + LE and MPA + hMG groups had comparable numbers of oocytes retrieved (17.5 ± 9.16 vs 16.8 ± 10.29, *p* > 0.05) and viable embryos (5.48 ± 3.25 vs 5.37 ± 4, *p* > 0.05). The hMG doses and durations of ovarian stimulation ((1949.89 ± 725.03 IU vs 2017.41 ± 653.32 IU and 9.03 ± 1.79 days vs 9.21 ± 2.18 days, respectively, *p* > 0.05) were similar between the two groups. The number of follicles with diameters larger than 10 or 14 mm on the trigger day was significantly higher in the study group (*p* < 0.01). To our surprise, there was a significantly lower mature oocyte rate (76.74 vs 83.77%, *p* < 0.01) and fertilization rate (79.23 vs 81.48%, *p* < 0.05) in the study group than in the control group. The rates of cycle cancellation for nonviable embryos did not differ between the two groups. No premature LH surge or moderate to severe OHSS was observed during the study (Table 2).

**TABLE 2:** Cycle characteristics of COS in the two groups.

Characteristic	Study group (hMG + MPA + LE; n = 224)	Control group (hMG + MPA; n = 224)	*p* Value
**hMG duration (d)**	9.03 ± 1.79	9.21 ± 2.18	0.33
**hMG dose (IU)**	1949.89 ± 725.03	2017.41 ± 653.32	0.3
**> 10-mm follicles on hCG day (n)**	23.17 ± 10	19.81 ± 9.23	<0.001
**> 14-mm follicles on hCG day (n)**	18.3 ± 9.32	13.84 ± 7.98	<0.001
**Percentage of women with profound pituitary suppression (%)**	7.14 (16/224)	14.29 (32/224)	0.015
**Punctured follicles (n)**	24.43 ± 0.86	23.8 ± 0.88	
**Oocyte retrieved (n)**	17.5 ± 9.16	16.81 ± 10.29	0.45
**Mature oocytes (n)**	13.45 ± 7.28	14.08 ± 9.22	0.420
**Fertilized oocytes (n)**	11.66 ± 6.47	12.54 ± 8.39	0.215
**Cleaved embryos (n)**	10.43 ± 5.78	11.29 ± 7.5	0.174
**High-quality embryos (n)**	4.75 ± 3.48	5.23 ± 4.73	0.22
**Blastocyst embryos (n)**	1.73 ± 2	1.53 ± 2.33	0.33
**All cryopreserved embryos (n)**	5.48 ± 3.25	5.37 ± 4	0.75
**Oocyte retrieval rate (%)**	71.98 (3,921/5,447)	71.27 (3,765/5,283)	0.409
**Mature oocyte rate (%)**	76.74 (3,009/3,921)	83.77 (3,154/3,765)	<0.001
**Fertilization rate (%)**	79.23 (2,384/3,009)	81.48 (2,570/3,154)	0.026
**Cleavage rate (%)**	97.86 (2,333/2,384)	98.40 (2,529/2,570)	0.157
**Viable embryo per oocyte retrieved (%)**	31.35 (1,230/3,924)	31.93 (1,202/3,765)	0.585
**Cycle cancellation rate (%)**	7.14 (16/224)	8 (18/224)	0.721

Data are presented as mean ± standard deviation or number (percentage). All the value of (n) were calculated per cycle. hMG, human menopausal gonadotropin; Oocyte retrieval rate was calculated according to all number of oocytes retrieved divided by all number of follicles punctured; Mature oocyte rate was count as the total number of mature oocytes divided by the total number of oocytes retrieved; Fertilization rate was calculated according to all number of fertilized oocytes divided by all number of mature oocytes; Cleavage rate was deemed to be the total number of cleavage embryos divided by the total number of fertilized oocytes; Viable embryo rate per oocyte was defined as the number of viable embryos divided by the number of oocytes retrieved. Cancellation rate was estimated based on the number of patients who had no viable embryos after complete oocyte retrieval.

### Hormone profiles during treatment

**FIGURE 2:**
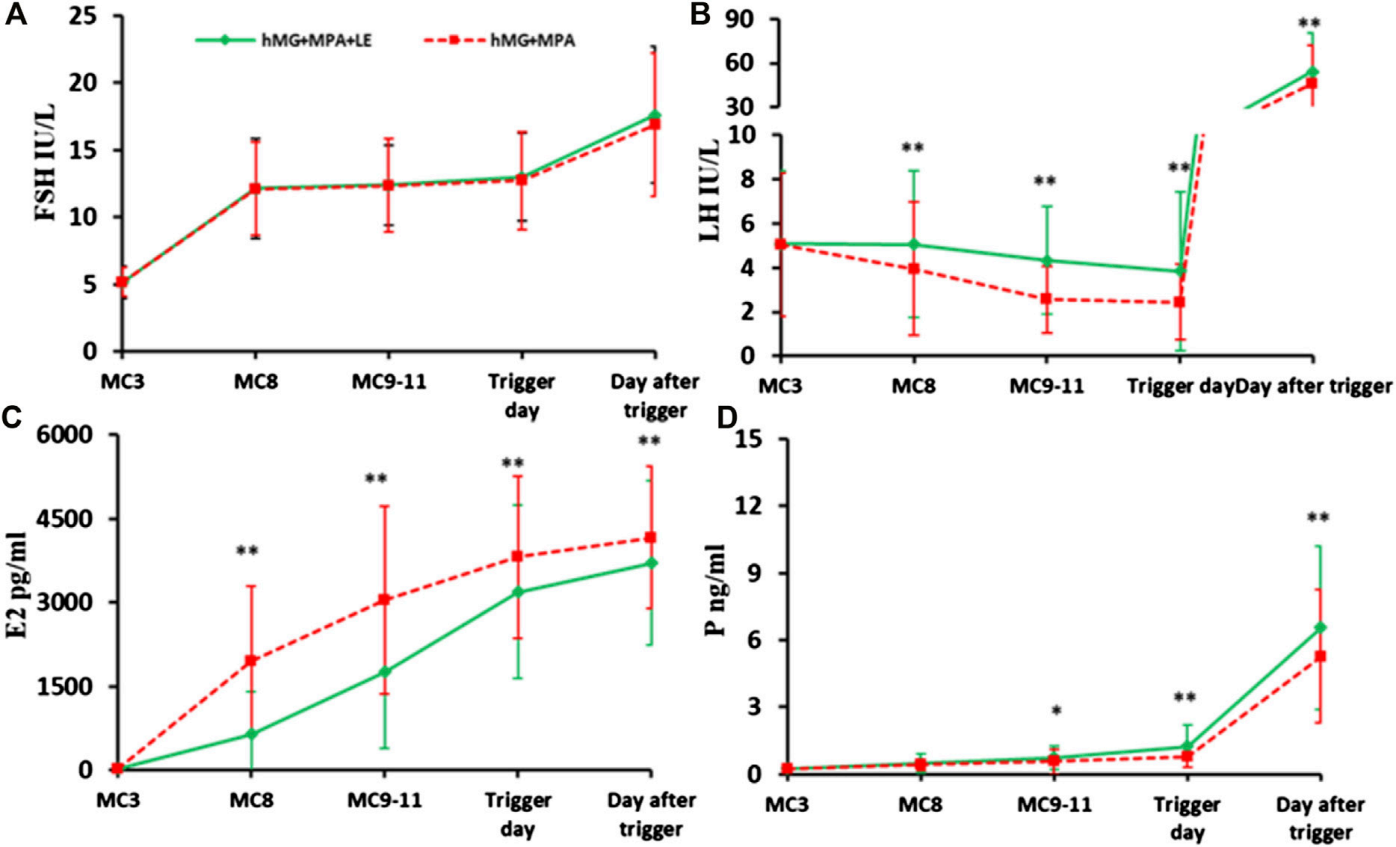
The dynamic changes in hormones during ovarian stimulation in the two groups. **(A)**. Serum FSH levels in the two groups during the COS; **(B)**. Serum LH concentration in the two groups during the COS; **(C)**. Serum E2 level between the two groups during the COS; **(D)**. Serum P levels in the two groups during the COS. The solid green lines represent the study group (hMG + MPA + LE) and the dotted red lines represent the control group (hMG + MPA). The asterisks de-note significant changes in hormone levels at the indicated time points (*p < 0.05, **p < 0.01).

The endocrine dynamics of FSH, LH, E2 and P4 during ovarian stimulation are presented in Figure 2. The FSH level increased dramatically after hMG administration for 5 days and then remained stable until the trigger day. After the dual trigger, the FSH level increased dramatically to nearly 20 mIU/ml. No significant differences were observed in FSH levels at any time point between the two groups (Figure 2A).

During the entire process of controlled ovarian stimulation (COS), LH in the two groups remained at a low level. However, there were differences in the change trend of LH between the two groups. LH in the control group showed a downwards trend. However, in the study group, LH remained basically unchanged in the first 5 days, followed by a downwards trend until the trigger day. The LH values were significantly higher in the study group than in the control group at each observation point during the COS (*p* < 0.01). In addition, significantly fewer patients experienced profound LH suppression in the study group (7.14%, 16/224) than in the control group (14.29%, 32/224, *p* < 0.05). None of the patients in either group experienced a premature LH surge (Figure 2B).

Serum E2 increased consistently with the development of multiple follicles and was significantly higher in the control group than in the study group at each observation point (*p* < 0.01) (Figure 2C).

In addition, the P level increased gradually during ovarian stimulation, especially on the day after trigger, in the two groups. On the trigger day and the day after, the *p* values in the study group were significantly higher than those in the control group (*p* < 0.01) (Figure 2D).

### Pregnancy outcomes after FET cycles

In this study, 382 women completed a total of 574 FET cycles: 189 patients completed 280 FET cycles in the MPA + hMG + LE group, and 193 patients completed 294 FET cycles in the MPA + hMG group. By the end of the study, 252 women had completed one FET cycle, 83 women had completed two FET cycles, 35 women had completed three FET cycles, nine women had completed four FET cycles, three women had completed five FET cycles, and the remaining 32 women did not complete FET cycles for personal reasons. A total of 970 embryos were thawed, and the rate of viable frozen-thawed embryos was 100%. The implantation rate in the study group (42.14%, 201/477) was significantly higher than that in the control group (34.69%, 171/493) (*p* < 0.05). The clinical pregnancy rate, miscarriage rate and ectopic pregnancy rate were comparable between the two groups (*p* > 0.05). The live birth rate and neonatal status require further statistical analysis (Table 3). The implantation rate in Table 3 represented the overall implantation rate and included all FET cycles associated with the IVF cycle. Similar calculations were used for other pregnancy outcome rates in this study. To minimize the impact of recurrent implantation failure and hysteroscopic surgery, we also analysed the implantation rate of first FET cycle in patients who underwent IVF for the first time. These supplementary data showed that the implantation rate in LE cotreatment group was significantly higher than that in the control group (57.4 vs 43.5%, *p* < 0.05) (Supplementary Table S1). To elucidate the effect of different endometrial preparation protocols on implantation rates during FET cycles, we performed sub-analyses of implantation rates for different endometrial preparation protocols in both groups. Our result showed nonsignificant difference of implantation rate among three types of endometrial preparation protocols in the two groups (Supplementary Table S2).

**TABLE 3:** Pregnancy outcomes of frozen-thawed embryos from the two groups.

Outcome	Study group (hMG + MPA + LE; n = 224)	Control group (hMG + MPA; n = 224)	*p* Value
Patients (n)	189	193	
FET cycles (n)	280	294	
Thawed embryos (n)	477	493	
Viable embryos after thawed (n)	477	493	
Endometrial preparation (n)			0.994
Natural cycle	47	49	
Mild stimulation	138	144	
Hormone therapy	95	101	
Endometrial thickness (mm)	10.4 ± 2.1	10.5 ± 2.1	0.894
Cumulate clinical pregnancy rate			
Per transfer (%)	54.29 (152/280)	49.32 (145/294)	0.234
Implantation rate (%)	42.14 (201/477)	34.69 (171/493)	0.017
Miscarriage rate (%)	7.89 (12/152)	8.97 (13/145)	0.074
Ectopic pregnancy rate (%)	1.32 (2/152)	2.07 (3/145)	0.678

Data are presented as mean ± standard deviation or number (percentage).

## Discussion

This retrospective cohort study was an endeavour to evaluate the potential role of letrozole as an adjuvant drug to improve the cycle outcomes of the standard PPOS protocol in infertile women with PCOS. In our preliminary findings, implantation rates in the combined group were superior to those in the PPOS group. Compared with those for the PPOS protocol alone, the number of oocytes and embryos obtained with letrozole combined with PPOS was similar, while the rates of mature and fertilized oocytes were significantly decreased. To our surprise, Gn consumption in the letrozole coadministration group did not reach statistical significance compared with that in the PPOS group.

The use of aromatase inhibitors in IVF patients remains controversial (Garcia-Velasco, 2012), and the data for letrozole cotreatment in high responders were quite limited in assisted reproductive technology cycles (Bulow et al., 2022). Our research demonstrated a superior implantation rate through letrozole cotreatment with the PPOS protocol in PCOS patients. It is worth noting that the implantation rate contained all FET cycles associated with the IVF cycle, patients with more than three FET cycles were also included. Women who failed pregnant in the first FET cycle routinely underwent hysteroscopy and endometrial curettage. We also analysed the implantation rate of first FET cycle in patients who underwent IVF for the first time to minimize the impact of recurrent implantation failure and hysteroscopic surgery. The supplementary data showed that the implantation rate in LE cotreatment group was significantly higher than that in the control group. A previous study reported contrary results, showing that letrozole supplementation had a harmful effect on pregnancy outcomes in vitro fertilization cycles in expected high responders (Yang et al., 2019). The conflicting results are due mainly to the premature progesterone rise during the late follicular phase in the fresh embryo cycle (Yang et al., 2019). The “freeze all” strategy in our study reduced the exposure of gametes and the endometrium to supraphysiological levels of progesterone (Yang et al., 2019). Another reason was that letrozole promotes the selection of dominant follicles, and embryos obtained from dominant follicles may be associated with improved implantation potential (Giorgetti et al., 1995; Hugues et al., 2005; Mermillod et al., 2008; Rosen et al., 2008; Gomez-Leon et al., 2020). Letrozole coadministration increases the secretion of LH by inhibiting the synthesis of oestrogen (Requena et al., 2008). Higher LH levels could probably advance the dominant follicle selection and drive growth of the dominant follicle after the beginning of diameter deviation (Hugues et al., 2005; Gomez-Leon et al., 2020). These fully developed preovulatory-stage follicles are more likely to yield high-quality oocytes (Rosen et al., 2008) and thus viable embryos (Mermillod et al., 2008). Previous studies indicated that embryos created from oocytes originating from the lead follicular group may associate with improved implantation potential, as they have less fragmentation (Giorgetti et al., 1995; Rosen et al., 2008). In addition, Huirne’s investigation demonstrated that smaller changes in LH may also raise the implantation rate (Huirne et al., 2005). In the present study, the mean LH level of the letrozole combined with the PPOS regimen seemed more stable and appropriate than that in the PPOS protocol alone. Nevertheless, contrary studies demonstrated that LH suppression by PPOS was associated with a higher implantation rate for patients with PCOS than with a short protocol (Chen et al., 2021). Hence, the optimal LH level required for the PPOS protocol in PCOS patients is worth exploring.

Interestingly, letrozole cotreatment was associated with decreased oocyte maturity and fertilization rates in comparison with standard PPOS protocols even though mature and fertilized oocyte yields were comparable. Previous investigations of letrozole coadministration reported conflicting results on oocyte maturity rate and fertilization rate (Oktay et al., 2006; Quinn et al., 2017; Jiang et al., 2022). Our findings are in accordance with the study published by Quinn et al. (Quinn et al., 2017) reporting that letrozole use was associated with a decreased maturity rate compared to the standard GnRH antagonist protocol in breast cancer patients and compared to those undergoing fertility preservation. The oocyte maturity rate is one of the predictors of the fertilization rate (Jawed et al., 2016); thus, a lower oocyte maturity rate may be accompanied by lower oocyte fertilization rate. Oktay et al. (Oktay et al., 2006) suggested that letrozole-containing cycles should trigger lead follicles with larger diameters than those of the traditional criteria, thus achieving better results. In addition to requiring disparate trigger criteria, letrozole cotreatment affects oocyte development by changing the follicular endocrine microenvironment. As an aromatase inhibitor, letrozole coadministration results in decreased oestrogen levels and the accumulation of precursors such as progesterone, testosterone and 17α-progesterone (Requena et al., 2008; Goldrat et al., 2015; Yang et al., 2021). These changes in the endocrine microenvironment may decrease calcium oscillations, consequently inhibiting oocyte cytoplasmic maturation, with effects on meiotic maturation contributing to reduced oocyte capacitation (part of its maturation) and thus fertilizability (Moor et al., 1980; Fukui et al., 1982; Qiao and Feng, 2011) in women with PCOS undergoing PPOS treatment. Letrozole combined with PPOS had an increased number of follicles >10 mm or >14 mm on the trigger day, as letrozole increased local levels of ovarian androgen and the expression of FSH receptor and insulin-like growth factor in ovarian granulosa cells, thereby recruiting more antral follicles into development (Dunaif, 1997; Requena et al., 2008). However, lower oocyte maturity and fertilization rates may eventually lead to similar numbers of mature and fertilized oocytes.

The continuous supply of progestin in the PPOS protocol can lead to strong pituitary suppression, presented as a sustained low level of LH during COS (Kuang et al., 2015), which was also demonstrated by the present study. After combined letrozole treatment, the LH level remained steady in the first 5 days of treatment and then slowly declined until the trigger day. Thus, the mean LH levels in the study group were higher than those in the control group during the entire process of COS, releasing the pituitary from the deep inhibition of progestin. This may be related to the biological mechanism of letrozole, which can block oestrogen biosynthesis, thereby reducing negative oestrogenic feedback to the hypothalamic/pituitary axis and increasing the LH level (Garcia-Velasco, 2012). Nevertheless, although pituitary suppression in the PPOS protocol was significantly reduced after letrozole coadministration, there was no significant difference in Gn consumption, which was unexpected, as previous studies suggested that letrozole appeared to have the potential to reduce the total gonadotropin dose required for ovarian stimulation (Dunaif, 1997; Requena et al., 2008; Garcia-Velasco, 2012). Contrary to the situation of low responders with lower expression of FSH receptor in the granulosa cells (Cai et al., 2007), high responders had FSH receptor overexpression in their follicular granulosa cell population, and this might be the reason letrozole could not reduce the total gonadotropin dose needed for ovarian stimulation in hyperresponders (Yang et al., 2019). In addition, the sample size in our study may not have been large enough to make the difference statistically significant.

PCOS patients are more likely to experience OHSS during IVF (Tshzmachyan and Hambartsoumian, 2020). It is notable that none of these patients experienced moderate or severe OHSS, although the average number of oocytes retrieved was more than 15. The application of a dual trigger is one of the main contributory factors to OHSS, as hCG has a prolonged half-life and sustained luteotropic activity, and reducing the hCG dosage may help reduce the occurrence of OHSS (Lu et al., 2016). Another key strategy is that all viable embryos be cryopreserved after ovarian stimulation for later thawing and transfer (Massin, 2017). This freeze-all policy avoids the deleterious effects of the supraphysiological hormonal milieu during COS. Beyond that, letrozole co-administration is highly effective in preventing OHSS in PCOS patients (Tshzmachyan and Hambartsoumian, 2020). Consequently, complete avoidance of OHSS is made possible even in the high-risk PCOS population.

### Limitations

In our study, there was a waiting period between oocyte retrieval and embryo transfer, as we used the freeze-all strategy, and 1,462 extra embryos were not transferred; therefore, we cannot show the complete pregnancy rate data. Moreover, some women were less than 3 months pregnant, and some had not yet given birth; thus, we cannot provide data on ongoing pregnancy and live birth rates. Additionally, this retrospective study may have potential unknown or unmeasured covariates, which may lead to incomplete or inexact matching and thus lower the robustness of the study findings. Furthermore, 80% of PCOS patients in China weigh in the normal range (Yang et al., 2022), so our data showed that women with PCOS were thinner than those in other studies, leading our conclusion may not apply to all types of PCOS patients. Prospective randomized controlled studies with larger sample sizes and basic research should be performed in the future.

## Conclusion

Our study indicates that letrozole supplementation in the PPOS protocol recruited more antral follicles into the COS process, with lower mature and fertilized oocyte rates, but the fully developed oocytes had high-quality development potential, manifested as a superior implantation rate in women with PCOS. Although letrozole coadministration significantly reduced pituitary suppression compared with the PPOS protocol, Gn consumption showed no significant difference.

## Data Availability

The original contributions presented in the study are included in the article/Supplementary Material, further inquiries can be directed to the corresponding authors.

## Abbreviations

AFC, antral follicle count; BMI, body mass index; FET, frozen embryo transfer; FSH, follicle stimulating hormone; hCG, human chorionic gonadotropin; ICSI, intracytoplastic sperm injection; IVF, *in vitro* fertilization; LE, letrozole; LH, luteinizing hormone; MPA, medroxyprogesterone acetate; OHSS, ovarian hyperstimulation; P4, progestin; PCOS, polycystic ovarian syndrome; PPOS, progestin-primed ovarian stimulation.

## References

[B1] Balen A. H. Morley L. C. Misso M. Franks S. Legro R. S. Wijeyaratne C. N. (2016). The management of anovulatory infertility in women with polycystic ovary syndrome: An analysis of the evidence to support the development of global WHO guidance. Hum. Reprod. Update 22 (6), 687–708. 10.1093/humupd/dmw025 27511809

[B2] Bulow N. S. Dreyer Holt M. Skouby S. O. Birch Petersen K. Englund A. L. M. Pinborg A. (2022). Co-Treatment with letrozole during ovarian stimulation for IVF/ICSI: A systematic review and meta-analysis. Reprod. Biomed. Online 44 (4), 717–736. 10.1016/j.rbmo.2021.12.006 35183444

[B3] Cai J. Lou H. Y. Dong M. Y. Lu X. E. Zhu Y. M. Gao H. J. (2007). Poor ovarian response to gonadotropin stimulation is associated with low expression of follicle-stimulating hormone receptor in granulosa cells. Fertil. Steril. 87 (6), 1350–1356. 10.1016/j.fertnstert.2006.11.034 17296182

[B4] Chen C. Yu S. Yu W. Yan Z. Jin W. Si J. (2021). Luteinizing hormone suppression by progestin-primed ovarian stimulation is associated with higher implantation rate for patients with polycystic ovary syndrome who underwent *in vitro* fertilization/intracytoplasmic sperm injection cycles: Comparing with short protocol. Front. Physiol. 12, 744968. 10.3389/fphys.2021.744968 35222055 PMC8874211

[B5] Cummins J. M. Breen T. M. Harrison K. L. Shaw J. M. Wilson L. M. Hennessey J. F. (1986). A formula for scoring human embryo growth rates in *in vitro* fertilization: Its value in predicting pregnancy and in comparison with visual estimates of embryo quality. J. Vitro Fert. Embryo Transf. 3 (5), 284–295. 10.1007/BF01133388 3783014

[B6] Dunaif A. (1997). Insulin resistance and the polycystic ovary syndrome: Mechanism and implications for pathogenesis. Endocr. Rev. 18 (6), 774–800. 10.1210/edrv.18.6.0318 9408743

[B7] Fukui Y. Fukushima M. Terawaki Y. Ono H. (1982). Effect of gonadotropins, steroids and culture media on bovine oocyte maturation *in vitro* . Theriogenology 18 (2), 161–175. 10.1016/0093-691x(82)90100-5 16725736

[B8] Garcia-Velasco J. A. (2012). The use of aromatase inhibitors in *in vitro* fertilization. Fertil. Steril. 98 (6), 1356–1358. 10.1016/j.fertnstert.2012.09.042 23062732

[B9] Giorgetti C. Terriou P. Auquier P. Hans E. Spach J. L. Salzmann J. (1995). Embryo score to predict implantation after *in-vitro* fertilization: Based on 957 single embryo transfers. Hum. Reprod. 10 (9), 2427–2431. 10.1093/oxfordjournals.humrep.a136312 8530679

[B10] Goldrat O. Gervy C. Englert Y. Delbaere A. Demeestere I. (2015). Progesterone levels in letrozole associated controlled ovarian stimulation for fertility preservation in breast cancer patients. Hum. Reprod. 30 (9), 2184–2189. 10.1093/humrep/dev155 26109617

[B11] Gomez-Leon V. E. Ginther O. J. Domingues R. R. Guimaraes J. D. Wiltbank M. C. (2020). Necessity for LH in selection and continued growth of the bovine dominant follicle. Reproduction 159 (5), 559–569. 10.1530/REP-19-0342 32053494

[B12] Hugues J. N. Soussis J. Calderon I. Balasch J. Anderson R. A. Romeu A. (2005). Does the addition of recombinant LH in WHO group II anovulatory women over-responding to FSH treatment reduce the number of developing follicles? A dose-finding study. Hum. Reprod. 20 (3), 629–635. 10.1093/humrep/deh682 15618252

[B13] Huirne J. A. van Loenen A. C. Schats R. McDonnell J. Hompes P. G. Schoemaker J. (2005). Dose-finding study of daily GnRH antagonist for the prevention of premature LH surges in IVF/ICSI patients: Optimal changes in LH and progesterone for clinical pregnancy. Hum. Reprod. 20 (2), 359–367. 10.1093/humrep/deh601 15567880

[B14] Jawed S. Rehman R. Ali M. A. Abdullah U. H. Gul H. (2016). Fertilization rate and its determinants in intracytoplasmic sperm injection. Pak. J. Med. Sci. 32 (1), 3–7. 10.12669/pjms.321.8329 27022334 PMC4795883

[B15] Jiang X. Jiang S. Diao H. Deng K. Zhang C. (2022). Progestin-primed ovarian stimulation protocol with or without letrozole for patients with normal ovarian reserve: A retrospective cohort study. J. Clin. Pharm. Ther. 47 (4), 469–476. 10.1111/jcpt.13567 34796515

[B16] Kuang Y. Chen Q. Fu Y. Wang Y. Hong Q. Lyu Q. (2015). Medroxyprogesterone acetate is an effective oral alternative for preventing premature luteinizing hormone surges in women undergoing controlled ovarian hyperstimulation for *in vitro* fertilization. Fertil. Steril. 104 (1), 62–70. 10.1016/j.fertnstert.2015.03.022 25956370

[B17] Kuang Y. Hong Q. Chen Q. Lyu Q. Ai A. Fu Y. (2014). Luteal-phase ovarian stimulation is feasible for producing competent oocytes in women undergoing *in vitro* fertilization/intracytoplasmic sperm injection treatment, with optimal pregnancy outcomes in frozen-thawed embryo transfer cycles. Fertil. Steril. 101 (1), 105–111. 10.1016/j.fertnstert.2013.09.007 24161646

[B18] Liu Y. Chen Q. Yu S. Wang Y. He W. Chang H. Y. (2018). Progestin-primed ovarian stimulation with or without clomiphene citrate supplementation in normal ovulatory women undergoing *in vitro* fertilization/intracytoplasmic sperm injection: A prospective randomized controlled trial. Clin. Endocrinol. 88 (3), 442–452. 10.1111/cen.13532 29247457

[B19] Lu X. Hong Q. Sun L. Chen Q. Fu Y. Ai A. (2016). Dual trigger for final oocyte maturation improves the oocyte retrieval rate of suboptimal responders to gonadotropin-releasing hormone agonist. Fertil. Steril. 106 (6), 1356–1362. 10.1016/j.fertnstert.2016.07.1068 27490046

[B20] Macklon N. S. Stouffer R. L. Giudice L. C. Fauser B. C. (2006). The science behind 25 years of ovarian stimulation for *in vitro* fertilization. Endocr. Rev. 27 (2), 170–207. 10.1210/er.2005-0015 16434510

[B21] Massin N. (2017). New stimulation regimens: Endogenous and exogenous progesterone use to block the LH surge during ovarian stimulation for IVF. Hum. Reprod. Update 23 (2), 211–220. 10.1093/humupd/dmw047 28062551

[B22] Mermillod P. Dalbies-Tran R. Uzbekova S. Thelie A. Traverso J. M. Perreau C. (2008). Factors affecting oocyte quality: Who is driving the follicle? Reprod. Domest. Anim. 43 (2), 393–400. 10.1111/j.1439-0531.2008.01190.x 18638152

[B23] Moor R. M. Polge C. Willadsen S. M. (1980). Effect of follicular steroids on the maturation and fertilization of mammalian oocytes. Development 56, 319–335. 10.1242/dev.56.1.319 6967508

[B24] Oktay K. Hourvitz A. Sahin G. Oktem O. Safro B. Cil A. (2006). Letrozole reduces estrogen and gonadotropin exposure in women with breast cancer undergoing ovarian stimulation before chemotherapy. J. Clin. Endocrinol. Metab. 91 (10), 3885–3890. 10.1210/jc.2006-0962 16882752

[B25] Qiao J. Feng H. L. (2011). Extra- and intra-ovarian factors in polycystic ovary syndrome: Impact on oocyte maturation and embryo developmental competence. Hum. Reprod. Update 17 (1), 17–33. 10.1093/humupd/dmq032 20639519 PMC3001338

[B26] Quinn M. M. Cakmak H. Letourneau J. M. Cedars M. I. Rosen M. P. (2017). Response to ovarian stimulation is not impacted by a breast cancer diagnosis. Hum. Reprod. 32 (3), 568–574. 10.1093/humrep/dew355 28122888

[B27] Requena A. Herrero J. Landeras J. Navarro E. Neyro J. L. Salvador C. (2008). Use of letrozole in assisted reproduction: A systematic review and meta-analysis. Hum. Reprod. Update 14 (6), 571–582. 10.1093/humupd/dmn033 18812422 PMC2569859

[B28] Rosen M. P. Shen S. Dobson A. T. Rinaudo P. F. McCulloch C. E. Cedars M. I. (2008). A quantitative assessment of follicle size on oocyte developmental competence. Fertil. Steril. 90 (3), 684–690. 10.1016/j.fertnstert.2007.02.011 18249377 PMC4624406

[B29] Rotterdam E. A.-S. P. C. W. G. (2004). Revised 2003 consensus on diagnostic criteria and long-term health risks related to polycystic ovary syndrome. Fertil. Steril. 81 (1), 19–25. 10.1016/j.fertnstert.2003.10.004 14711538

[B30] Teede H. J. Misso M. L. Costello M. F. Dokras A. Laven J. Moran L. (2018). Recommendations from the international evidence-based guideline for the assessment and management of polycystic ovary syndrome. Hum. Reprod. 33 (9), 1602–1618. 10.1093/humrep/dey256 30052961 PMC6112576

[B31] Thessaloniki E. A.-S. P. C. W. G. (2008). Consensus on infertility treatment related to polycystic ovary syndrome. Hum. Reprod. 23 (3), 462–477. 10.1093/humrep/dem426 18308833

[B32] Timmons D. Montrief T. Koyfman A. Long B. (2019). Ovarian hyperstimulation syndrome: A review for emergency clinicians. Am. J. Emerg. Med. 37 (8), 1577–1584. 10.1016/j.ajem.2019.05.018 31097257

[B33] Tshzmachyan R. Hambartsoumian E. (2020). The role of Letrozole (LE) in controlled ovarian stimulation (COS) in patients at high risk to develop ovarian hyper stimulation syndrome (OHSS). A prospective randomized controlled pilot study. J. Gynecol. Obstet. Hum. Reprod. 49 (2), 101643. 10.1016/j.jogoh.2019.101643 31563697

[B34] Wang Y. Chen Q. Wang N. Chen H. Lyu Q. Kuang Y. (2016). Controlled ovarian stimulation using medroxyprogesterone acetate and hMG in patients with polycystic ovary syndrome treated for IVF: A double-blind randomized crossover clinical trial. Med. Baltim. 95 (9), e2939. 10.1097/MD.0000000000002939 PMC4782886 26945402

[B35] Xu X. Pommier S. Arbov T. Hutchings B. Sotto W. Foxcroft G. R. (1998). *In vitro* maturation and fertilization techniques for assessment of semen quality and boar fertility. J. Anim. Sci. 76 (12), 3079–3089. 10.2527/1998.76123079x 9928613

[B36] Yang A. M. Cui N. Sun Y. F. Hao G. M. (2021). Letrozole for female infertility. Front. Endocrinol. 12, 676133. 10.3389/fendo.2021.676133 PMC8245002 34220713

[B37] Yang R. Li Q. Zhou Z. Qian W. Zhang J. Wu Z. (2022). Changes in the prevalence of polycystic ovary syndrome in China over the past decade. Lancet Reg. Health. West. pac. 25, 100494. 10.1016/j.lanwpc.2022.100494 35669932 PMC9162959

[B38] Yang X. Lin G. Lu G. Gong F. (2019). Letrozole supplementation during controlled ovarian stimulation in expected high responders: A pilot randomized controlled study. Reprod. Biol. Endocrinol. 17 (1), 43. 10.1186/s12958-019-0483-x 31077214 PMC6511177

