# Supplementary material for: Letrozole cotreatment with progestin-primed ovarian stimulation in women with polycystic ovary syndrome undergoing IVF treatment
Source: Front Physiol. 2022 Aug 19;13:965210. doi: 10.3389/fphys.2022.965210 (PMC9437256; doi:10.3389/fphys.2022.965210)
Supplement: Supplementary file 1 [file Table1.DOCX]

**Supplementary Table 1. Pregnancy outcomes of patient underwent the first cycle of IVF and transplantation from the two groups.**

| **Outcome** | **Study group** | **Control group** | **P value** |
| --- | --- | --- | --- |
|  | **(hMG+MPA+LE)** | **(hMG+MPA)** |  |
| Patients (n) | 161 | 173 |  |
| FET cycles (n) | 161 | 173 |  |
| Thawed embryos (n) | 223 | 260 |  |
| Viable embryos after thawed (n) | 223 | 260 |  |
| Clinical pregnancy rate |  |  |  |
| Per cycle (%) | 66.5 (107/161) | 59.5 (103/173) | 0.191 |
| Implantation rate (%) | 57.4 (128/223） | 43.5(113/260) | 0.002 |

Note: Data are presented as number (percentage).
