# Supplementary material for: Letrozole cotreatment with progestin-primed ovarian stimulation in women with polycystic ovary syndrome undergoing IVF treatment
Source: Front Physiol. 2022 Aug 19;13:965210. doi: 10.3389/fphys.2022.965210 (PMC9437256; doi:10.3389/fphys.2022.965210)
Supplement: Supplementary file 2 [file Table2.DOCX]

**Supplementary Table 2. The implantation rate among three types of endometrial lining preparation in both groups.**

| **Outcome** |  | **Natural cycle** | **Mild**  **stimulation** | **Hormone therapy** | **P value** |
| --- | --- | --- | --- | --- | --- |
| **Study group** | FET cycles (n) | 47 | 138 | 95 |  |
|  | Thawed embryos (n) | 80 | 235 | 163 |  |
|  | Viable embryos after thawed (n) | 80 | 235 | 163 |  |
|  | Implantation rate (%) | 35 (28/80) | 44.7 (105/235) | 42 (68/162) | 0.371 |
| **Control group** | FET cycles (n) | 49 | 144 | 101 |  |
|  | Thawed embryos (n) | 84 | 240 | 169 |  |
|  | Viable embryos after thawed (n) | 84 | 240 | 169 |  |
|  | Implantation rate (%) | 31 (26/84) | 35.8 (86/240) | 34.9 (59/169) | 0.719 |

Note: Data are presented as number (percentage).
