# Supplementary material for: Letrozole cotreatment with progestin-primed ovarian stimulation in women with polycystic ovary syndrome undergoing IVF treatment
Source: Front Physiol. 2022 Aug 19;13:965210. doi: 10.3389/fphys.2022.965210 (PMC9437256; doi:10.3389/fphys.2022.965210)
Supplement: Supplementary file 3 [file Table3.DOCX]

**Supplementary table 3. Baseline characteristics of women undergoing IVF/ICSI before marching.**

| **Characteristic** | **Study group** | **Control group** | **P value** |
| --- | --- | --- | --- |
|  | **(hMG+MPA+LE; n=256)** | **(hMG+MPA; n=2197)** |  |
| **ge (y), mean ± SD** | 31.66±3.59 | 34.14±3.39 | 0 |
| **Duration of infertility (y), mean ± SD** | 3.73±2.44 | 3.57±2.13 | 0.32 |
| **Primary infertility, n (%)** | 66 (169/256) | 64.5 (1417/2197) | 0.02 |
| **Previous IVF failure, n (%)** |  |  | 0 |
| **0** | 195 | 1301 |  |
| **1–2** | 38 | 582 |  |
| **> 3** | 23 | 314 |  |
| **BMI (kg/m2), mean ± SD** | 24.35±4.44 | 22.62±3.5 | 0 |
| **FSH (IU/L)** | 5.18±1.23 | 5.22±1.23 | 0.66 |
| **LH (IU/L)** | 5.24±3.51 | 4.61±2.75 | 0.01 |
| **E_2_ (pg/mL)** | 35.86±12.74 | 33.51±12.59 | 0 |
| **P (ng/mL)** | 0.24±0.13 | 0.27±0.13 | 0 |
| **AFC** | 21.44±7.61 | 19.07±4.82 | 0 |

Note: Data are presented as mean ± standard deviation or number (percentage).
